# Reduced burden of diabetes and improved quality of life: Experiences from unrestricted day‐and‐night hybrid closed‐loop use in very young children with type 1 diabetes

**DOI:** 10.1111/pedi.12872

**Published:** 2019-06-13

**Authors:** Gianluca Musolino, Klemen Dovc, Charlotte K. Boughton, Martin Tauschmann, Janet M. Allen, Katrin Nagl, Maria Fritsch, James Yong, Emily Metcalfe, Dominique Schaeffer, Muriel Fichelle, Ulrike Schierloh, Alena G. Thiele, Daniela Abt, Harald Kojzar, Julia K. Mader, Sonja Slegtenhorst, Nicole Ashcroft, Malgorzata E. Wilinska, Judy Sibayan, Nathan Cohen, Craig Kollman, Sabine E. Hofer, Elke Fröhlich‐Reiterer, Thomas M. Kapellen, Carlo L. Acerini, Carine de Beaufort, Fiona Campbell, Birgit Rami‐Merhar, Roman Hovorka, on behalf of Kidsap Consortium

**Affiliations:** ^1^ Wellcome Trust‐MRC Institute of Metabolic Science University of Cambridge Cambridge UK; ^2^ Department of Paediatric Endocrinology, Diabetes and Metabolic Diseases University Children's Hospital, University Medical Centre Ljubljana Slovenia; ^3^ Department of Paediatrics University of Cambridge Cambridge UK; ^4^ Department of Pediatrics and Adolescent Medicine Medical University of Vienna Vienna Austria; ^5^ Department of Paediatric Diabetes Leeds Children's Hospital Leeds UK; ^6^ Department of Pediatric Diabetes and Endocrinology Clinique Pédiatrique, Centre Hospitalier Luxembourg City Luxembourg; ^7^ Division for Paediatric Diabetology University of Leipzig Leipzig Germany; ^8^ Department of Pediatrics I Medical University of Innsbruck Innsbruck Austria; ^9^ Department of Internal Medicine, Division of Endocrinology and Diabetology Medical University of Graz Graz Austria; ^10^ Department of Nutrition & Dietetics Cambridge University Hospitals NHS Foundation Trust Cambridge UK; ^11^ Jaeb Center for Health Research Tampa Florida; ^12^ Department of Pediatrics and Adolescent Medicine Medical University of Graz Graz Austria; ^13^ Department of Pediatrics Free University VUB Brussels Belgium

**Keywords:** artificial pancreas, closed‐loop insulin delivery, type 1 diabetes, very young children

## Abstract

**Objective:**

To evaluate the experiences of families with very young children aged 1 to 7 years (inclusive) with type 1 diabetes using day‐and‐night hybrid closed‐loop insulin delivery.

**Methods:**

Parents/caregivers of 20 children aged 1 to 7 years with type 1 diabetes completed a closed‐loop experience survey following two 3‐week periods of unrestricted day‐and‐night hybrid closed‐loop insulin therapy using Cambridge FlorenceM system at home. Benefits, limitations, and improvements of closed‐loop technology were explored.

**Results:**

Responders reported reduced burden of diabetes management, less time spent managing diabetes, and improved quality of sleep with closed‐loop. Ninety percent of the responders felt less worried about their child's glucose control using closed‐loop. Size of study devices, battery performance and connectivity issues were identified as areas for improvement. Parents/caregivers wished for more options to input information to the system such as temporary glucose targets.

**Conclusions:**

Parents/caregivers of very young children reported important quality of life benefits associated with using closed‐loop, supporting adoption of this technology in this population.

## 1 INTRODUCTION

The burden of diabetes management in very young children is a significant challenge for caregivers. In this vulnerable population glycemic control is particularly demanding, with unpredictable eating habits, irregular bouts of physical activity, erratic behavior, and significant fear of hypoglycemia. The incidence of hypoglycemia is high in children <6 years of age, with most cases occurring during the night.^1^ Caregivers of very young children with type 1 diabetes often routinely monitor blood glucose levels overnight, with consequent sleep disruption and anxiety.^2^ Significant fear of hypoglycemia leads to reduced quality of life and sub‐optimal glycemic control.^3^

Closed‐loop systems, which automatically and continuously adjust insulin delivery according to real‐time sensor glucose levels, may reduce burden of diabetes management. A recent trial involving very young children aged 1 to 7 years demonstrated that unrestricted home use of day‐and‐night closed‐loop is feasible and safe in managing glucose control.^4^

The psychosocial benefits of closed‐loop have been explored in older children, adolescents, and adults^5^, ^6^, ^7^ but the impact of closed‐loop use in very young children with type 1 diabetes is yet to be assessed.

The aim of this study was to evaluate the experience of families with very young children with type 1 diabetes using day‐and‐night hybrid closed‐loop system during unrestricted living.

## 2 METHODS

Twenty‐four children aged 1 to 7 years (inclusive), with type 1 diabetes for at least 6 months, using insulin pump therapy for at least 3 months and with glycated hemoglobin (HbA1c) ≤ 97 mmol/mol (11%) were recruited into a study adopting an open‐label, multicenter, multinational, randomized, two‐period crossover design in the UK (Cambridge and Leeds), Germany (Leipzig), Luxembourg (DECCP/Clinique Pédiatrique/CH de Luxembourg), and Austria (Graz, Innsbruck and Vienna).

Participants underwent two 3‐week periods of hybrid closed‐loop use comparing standard strength insulin aspart (U100; Novo Nordisk, Bagsvaerd, Denmark) with diluted insulin aspart (U20; Novo Nordisk), separated by a 1‐4 week washout. During the 2‐4 week run‐in period, participants were trained on the study insulin pump and real‐time continuous glucose sensor. At the end of run‐in period compliance was assessed. Participants and caregivers received training on the closed‐loop system after run‐in; competency was assessed. During the study period participants were free to consume meals of their choice and no restrictions were imposed on traveling. Caregivers were encouraged to use closed‐loop during physical activity and to announce these periods to the control algorithm. Meal bolus calculations were performed using the pump bolus calculator. Further details for the study design can be found in the main manuscript of the trial.^4^

The trial was approved by independent Research Ethics Committees and regulatory authorities in the UK, Luxembourg, Germany, and Austria and in accordance with Declaration of Helsinki. Parents/guardians of participants signed informed consent before study related activities were initiated. Whenever possible, and in line with recommendations by local ethics committees, assent of study participants was obtained in addition to the consent of the parents/guardians or legal representatives.

A modified 640G insulin pump (investigational use only; Medtronic, Northridge, California) and real‐time continuous glucose sensor (Enlite 3, Medtronic) were used during the two intervention treatment periods. The FlorenceM closed‐loop system (Supporting Information, Figure S1) utilized a model predictive control algorithm (version 0.3.46, University of Cambridge, Cambridge, UK) hosted on an Android smartphone (Galaxy S4, Samsung, South Korea).

At the end of the study, caregivers were asked to complete the Closed‐loop Experience Questionnaire (Table S1), consisting of two parts. Part A lists six questions about closed‐loop experience during the study using a numerical scale from 1 to 5, from “Strongly Agree” to “Strongly Disagree.” For each answer a mean score was calculated. Questions are reverse scored (except for question 3), so a higher score denotes more satisfaction with the closed‐loop system. Part B consists of three open questions regarding perceived benefits and limitations of the system with space for suggestions for additional features. Quotes in French and German were translated in English prior to analysis. Trial registration NCT03101865 (ClinicalTrials.gov)

## 3 RESULTS

Completed surveys were available for 20 of 24 enrolled participants: seven from Austria, two from Germany, four from Luxembourg, and seven from UK (Table S2). Baseline characteristics of the participants who completed the survey are shown in Table S3. There was no difference in HbA1c of study participants at enrolment between responders and non‐responders (mean HbA1c 7.4% vs 7.4%, 57 mmol/mol vs 57 mmol/mol). The results of Part A of the Closed‐loop Experience Questionnaire are presented in Table 1. The median Closed‐loop Experience Survey total score was 28 out of 30 (IQR 26‐29).

**Table 1:** Closed‐loop Experience Questionnaire. Summary of outcomes of Part A (*N* = 20)

		Strongly agree				Strongly disagree
	Mean score^a^	1	2	3	4	5
**During the 6‐week intervention…**						
Q1. I was happy to have my child's glucose levels controlled automatically by the system.^b^	4.8	80%	15%	5%	0%	0%
Q2. I spent less time to manage my child's diabetes (glucose testing, adjusting insulin therapy, keeping a diary, data review…).^b^	4.2	40%	45%	10%	5%	0%
Q3. Using the system took more time and work than it is worth.	4.5	0%	0%	5%	40%	55%
Q4. I was less worried about my child's glucose control.^b^	4.4	45%	45%	10%	0%	0%
Q5. I had less trouble sleeping.^b^	4.5	65%	25%	5%	5%	0%
Q6. I would recommend closed‐loop to others.^b^	4.9	90%	10%	0%	0%	0%

a Scale 1‐5. Higher score denotes more satisfaction with the CL system.

b Questions have been reverse scored so a higher score denotes more satisfaction with the CL system.

### 3.1 Part A

#### 3.1.1 Overall experience

Ninety percent of caregivers would strongly recommend the system to others. After just 6 weeks of use, 95% of responders were satisfied with their child's glucose levels being controlled automatically by the system, suggesting rapid gain of trust in the closed‐loop.

#### 3.1.2 Reducing the burden of diabetes

Caregivers acknowledged closed‐loop reduced the burden of diabetes management. Eighty‐five percent of the responders reported spending less time managing their child's diabetes (finger‐pricks, insulin therapy adjustment, regular data review) with closed‐loop. One caregiver (5%) felt that closed‐loop took more time and work than it was worth.

#### 3.1.3 Reduced stress levels

Ninety percent of responders reported that they were less worried about their child's glucose control when using closed‐loop. Eighteen out of 20 (90%) caregivers reported having less trouble sleeping whilst their child was using the system.

### 3.2 Part B

#### 3.2.1 Perceived benefits

Responders identified several clinical benefits of closed‐loop regarding reduced hypoglycemic events (“*…safe system with little hypoglycemia*,” “*…no hypos at night*”) and more stable glycemic control (“*more sugar stability*,” “*…Values more stable throughout the day*”), especially during night‐time (“*…little fluctuation especially at night*,” “*The overnight control was excellent*,” “*We have had issues with overnight and the system helped*”*)*.

Caregivers were positive about the system's responsiveness with its “*automatic basal rate adjustment for high and low glucose levels*” and “*automatic shutdown in case of hypoglycemia*.” They also felt reassured by the ability of the closed‐loop system to “*give the exact amount of insulin which is required*.” These features reduced the burden of disease management as caregivers spent less time performing diabetes‐related activities *(*“*less ‘visual inspection’ on pump + less BG measurements*”). Real‐time visualization of data (“*to see the pattern on a big screen*” and the “*amount of data for analysis/decision making*”) was highlighted as a very useful benefit.

Responders emphasized the improvement in quality of sleep with closed‐loop (“*the quieter nights*”) both for caregivers (“*first time we as parents were able to sleep the night straight since diagnosis*”*)* and users (“*Also…our child…was able to sleep undisturbed*”*)*.

Many caregivers felt reassured (“*Having the system working at school also for us was reassuring*”) and less stressed (“*Less worry*, *better quality of life*,” “*I was less worried about blood sugar*”). Some caregivers suggested that closed‐loop had a positive impact on their child's well‐being (“*Overall we noticed the effect on our child's life: he had a significant improvement in developing his walking & talking*,” “*It has made a massive difference to the last few months. My son was a lot more confident*, *less angry and generally happier*”*)*.

#### 3.2.2 Limitations of the closed‐loop system

Several caregivers highlighted limitations of the system relating to the size of the study devices (“*The device was bulky*,” “*phone handset a little too large*”), battery performance (“*phones need to be charged a lot*”), and connectivity issues (“*the systems…would lose connection even when they were within a reasonable range e.g. in the same room*”).

While alarms were perceived as intrusive (“*a curse*”) by some users, and seemed to create some “*anxiety*” due to the “*noise from the phone*” and “*warning vibration from the pump*,” for others they were considered reassuring (“*The alarms were…a blessing…and created… reassurance to ourselves*”).

#### 3.2.3 Features to be implemented

Responders were invited to suggest additional features for the system. These included mostly technical improvements: “*a longer range*,” “*the closed‐loop system incorporated into the pump*,” “*be able to access blood sugars from different phones*,” or “*bolusing without having to touch the pump*.” Some participants also suggested more user‐adjustable settings and the option to be able to override the closed‐loop system: “*ability to suggest change of closed‐loop action if desired*,” “*more variable control*, *like OpenAPS e.g. temp targets*, *micro bolus for food*, *ability to change settings throughout the trial*.”

Some caregivers also wanted an option to incorporate into the system additional information regarding food types in order to improve its effectiveness (“*Use phone to input food type*, *e.g. spaghetti*, *pizza etc*.”*)*.

## 4 DISCUSSION

This survey reports on the acceptability of closed‐loop use in caregivers of very young children with type 1 diabetes in the home setting. The strengths of this study lie in having collected perspectives of users from different European countries. Participants used the system without remote monitoring or close supervision, therefore assessing real‐world use and supporting generalizability of study findings. A previous study using closed‐loop in similar aged children was of very short duration and in a highly supervised setting.^8^

This study identifies important positive glycemic and quality of life benefits of closed‐loop reported by caregivers. The perceived benefit of nocturnal control achieved by this technology reduced caregivers' anxiety, and improved sleep quality. The system was also able to reduce the burden of diabetes by decreasing the amount of time spent on day to day diabetes management.

There are some limitations to our study. Closed‐loop experience was assessed after a relatively short period of use and in a limited number of caregivers without a control group. There is a potential selection bias, as more highly motivated families are likely to participate in closed‐loop trials. The reasons why four caregivers (including the caregiver of the participant who withdrew from the study) did not complete the survey were not recorded. We did not collect information regarding the demographics of parents/caregivers (age, gender, socio‐economic status). Therefore, other possible differences between the participants whose caregivers responded to the survey and those who did not, have not been evaluated. Use of self‐reported questionnaires also has limitations. The Closed‐loop Experience Questionnaire has not yet been validated. Given the very good glycemic control of the participants enrolled (Table S3), it is possible that the study population is not representative of the broader population of young children with type 1 diabetes. We need to verify if the reported benefits would result in similar findings in a less selective population. We aim to address these issues in a longer duration follow‐up study (NCT03784027, ClinicalTrials.gov). As part of the planned study caregivers will take part in semi‐structured interviews in order to gather their experiences using the closed‐loop system and the quality of life impact.

There is significant interest in automated insulin delivery systems for caregivers of children with type 1 diabetes. One expression of this is the growing patient‐designed and ‐driven artificial pancreas systems, the so‐called DIY (Do‐it‐Yourself) community.^9^ The desire from some caregivers to be more involved in interacting with the system was evident in some responses to the questionnaire. Future developments of this technology should provide users with a wider range of adjustable settings to improve user experience.

Psychosocial perspectives of closed‐loop system use from both users and caregivers are vital. User feedback is instructive to help the manufacturers and the researchers improve the systems and develop additional features to be implemented. If closed‐loop is to be widely adopted as standard management of type 1 diabetes in the future, it is essential for healthcare providers to explore users' real‐life experience using this technology in order to meet their expectations properly.

In conclusion, results from our survey undertaken by caregivers of very young children with type 1 diabetes demonstrated overall satisfaction with unrestricted hybrid closed‐loop use. Future closed‐loop systems may address some of the identified limitations. Further studies of longer duration are required to better understand closed‐loop experience in this age group.

## CONFLICT OF INTEREST

M.T. reports having received speaker honoraria from Minimed Medtronic and Novo Nordisk. M.Fr. has received speaker honoraria from Minimed Medtronic and has served on advisory boards for Eli Lilly. J.K.M. is a member in the advisory board of Becton‐Dickinson, Boehringer Ingelheim, Eli Lilly, Medtronic and Sanofi, and has received speaker honoraria from Abbott Diabetes Care, Astra Zeneca, Eli Lilly, Nintamed, Novo Nordisk, Roche Diabetes Care, Sanofi, Servier, and Takeda. M.E.W. has received license fees from Becton‐Dickinson and has served as a consultant to Beckton‐Dickinson. S.E.H. declares speaker honoraria from Eli Lilly and Sanofi. E.F.R. reports having received speaker honoraria from Minimed Medtronic and Eli Lilly, serving on advisory boards for Eli Lilly. T.M.K. has received speaker honoraria from Minimed Medtronic, Roche, and Eli Lilly and is member of an advisory board for Abbott Diabetes Care. C.dB. has received speaker honoraria from Minimed Medtronic, and is member of their European Psychology Advisory Board. F.C. does attend Advisory Boards and obtain speaking fees for Abbott, Medtronic, Lilly, and NovoNordisk. B.R.M. reports having received speaker honoraria from Minimed Medtronic, Eli Lilly, Roche, Menarini, and Novo Nordisk, serving on advisory boards for Eli Lilly. R.H. reports having received speaker honoraria from Minimed Medtronic, Eli Lilly, BBraun, and Novo Nordisk, serving on advisory panel for Eli Lilly, receiving license fees from BBraun and Medtronic; and having served as a consultant to BBraun, Sanofi‐Aventis, and Profil. R.H. and M.E.W. report patents and patent applications. G.M., K.D., C.B., J.M.A., K.N., J.Y., E.M., D.S., M.Fi., U.S., A.G.T., D.A., H.K., S.S., N.A., J.S., N.C., C.K., and C.L.A. declare no competing financial interests exist.

## AUTHOR CONTRIBUTIONS

R.H. and N.A. coordinated the study. R.H., S.E.H., E.F.R., T.M.K., C.L.A., C.dB., F.C., B.R.M., J.K.M, M.E.W., and M.T. designed the study. M.T., J.M.A., K.N., M.Fr., J.Y., E.M., D.S., M.Fi., U.S., A.G.T., D.A., S.S., S.E.H., E.F.R., T.M.K., C.L.A., C.dB., F.C., and B.R.M. screened and enrolled participants and arranged informed consent from the participants. R.H. designed and implemented the glucose controller. G.M., K.D., C.B., and R.H. interpreted the results and wrote the manuscript. All authors critically reviewed the report. No writing assistance was provided.

## Supporting information


**FIGURE S1** FlorenceM closed‐loop system prototype
**TABLE S1.** Closed‐loop experience questionnaire
**TABLE S2.** Number of subjects completing closed‐loop parent experience questionnaire
**TABLE S3.** Participant characteristics at randomization
