# Supplementary material for: Reduced burden of diabetes and improved quality of life: Experiences from unrestricted day‐and‐night hybrid closed‐loop use in very young children with type 1 diabetes
Source: Pediatr Diabetes. 2019 Jun 13;20(6):794–9. doi: 10.1111/pedi.12872 (PMC6771658; doi:10.1111/pedi.12872)
Supplement: Supplementary file 1 — FIGURE S1 FlorenceM closed‐loop system prototype TABLE S1. Closed‐loop experience questionnaire TABLE S2. Number of subjects completing closed‐loop parent experience questionnaire TABLE S3. Participant characteristics at randomization [file PEDI-20-794-s001.docx]

**Supplementary Appendix**

**Table of contents**

**KidsAP Consortium**

**Figure S1.** FlorenceM closed-loop system prototype

**Table S1.** Closed-loop Experience Questionnaire

**Table S2.** Number of subjects completing closed-loop parent experience questionnaire

**Table S3.** Participant characteristics at randomization

**KidsAP Consortium**

The KidsAP Consortium is composed by the following investigators:

*University of Cambridge, UK*: M Tauschmann, JM Allen, C Boughton, G Musolino, ME Wilinska, Y Ruan, S Slegtenhorst, S Hartnell, CL Acerini, R Hovorka

*Medical University of Graz, Graz, Austria:* H Kojzar, JK Mader, E Suppan, EE Fröhlich-Reiterer

*DECCP, Clinique Pédiatrique / CH de Luxembourg, Luxembourg:* D. Schaeffer, M. Fichelle, U Schierloh, C. de Beaufort

*University of Leipzig, Leipzig, Germany:* AG Thiele, H Bartelt, TM Kapellen

*Medical University of Innsbruck, Innsbruck, Austria:* D Abt, D Meraner, E Binder, L Hackl, E Steichen, SE Hofer

*Leeds Children's Hospital, UK*: J Exall, J Yong, E Metcalfe, F Campbell

*Medical University of Vienna, Vienna, Austria:* B Rami Merhar, M Fritsch, K Nagl, S Katzenbeisser-Pawlik

*University of Edinburgh, Edinburgh, UK*: J Lawton

*HEVA HEOR SARL, Lyon, France*: S Roze

*Jaeb Center for Health Research, Tampa, FL, USA*: J Sibayan, C Kollman, N Cohen, RW Beck

*Diasend AB, Askim, Sweden*: A Sonesson, D Stjern, R Laurits, T Jansson

Figure S1. FlorenceM closed-loop system prototype

(A) The system combines a continuous glucose monitoring transmitter with Enlite 3 sensor (Medtronic), an insulin pump (modified 640G pump, investigational use only, Medtronic), and a smartphone (Galaxy S4, Samsung) with the control algorithm (Cambridge). The mobile phone communicated wirelessly with the study pump through a proprietary translator device included in the smartphone’s enclosure (investigational use only). By using the information received from the glucose sensor, every ten minutes the algorithm computes the insulin infusion rate to be delivered by the study insulin pump. (B) Photo of a UK participant (obtained with consent), and (C) photo of an Austrian subject (obtained with consent) using the system.


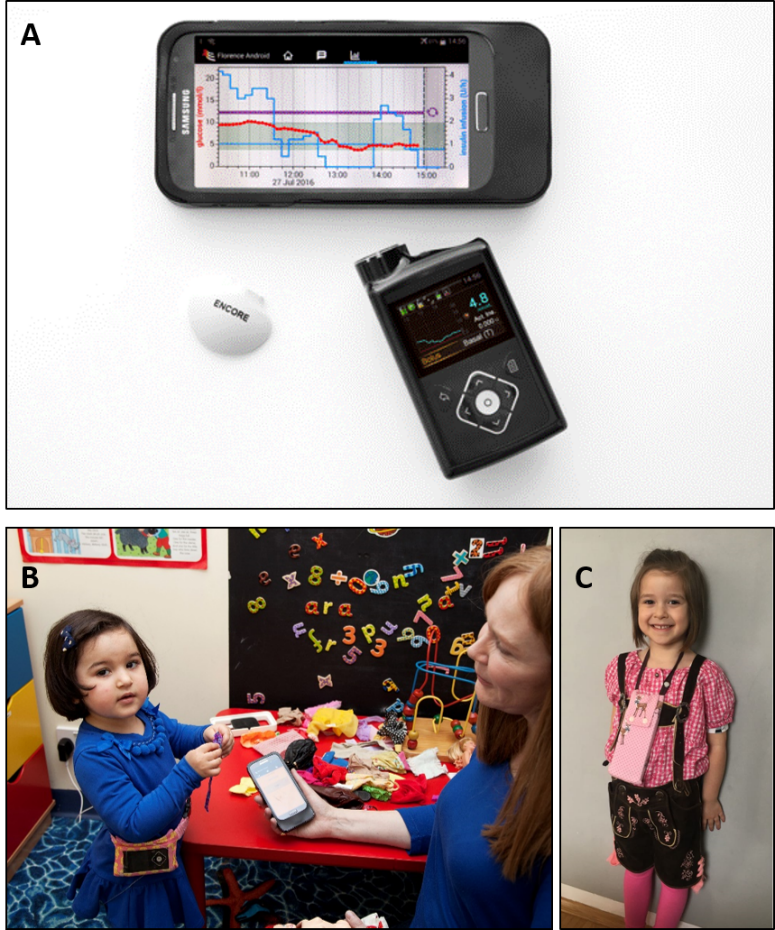


**Table S1. Closed-loop Experience Questionnaire**

**Part A**

| **During the 6-week intervention…** | | **Strongly Agree** |  |  |  | **Strongly Disagree** |
| --- | --- | --- | --- | --- | --- | --- |
| Q1. | I was happy to have my child’s glucose levels controlled automatically by the system | 1 | 2 | 3 | 4 | 5 |
| Q2. | I spent less time to manage my child’s diabetes (glucose testing, adjusting insulin therapy, keeping a diary, data review…) | 1 | 2 | 3 | 4 | 5 |
| Q3 | Using the system took more time and work than it is worth | 1 | 2 | 3 | 4 | 5 |
| Q4. | I was less worried about my child’s glucose control | 1 | 2 | 3 | 4 | 5 |
| Q5. | I had less trouble sleeping | 1 | 2 | 3 | 4 | 5 |
| Q6. | I would recommend Closed-Loop to others | 1 | 2 | 3 | 4 | 5 |
| **Part B** | | | | | | |
| Q6. What did you like about the Closed-Loop system? | | | | | | |
| Q7. What are the things you did not like about the system? | | | | | | |
| Q8. Would you like the Closed-Loop system to have additional features? If yes, which ones? | | | | | | |

## Table S2. Number of subjects completing closed-loop parent experience questionnaire

| **Country** | **Number of participants** |
| --- | --- |
| **Austria** | **7** |
| - Vienna | 4 |
| - Innsbruck | 2 |
| - Graz | 1 |
| **Germany** (Leipzig) | **2** |
| **Luxembourg** (Luxembourg City) | **4** |
| **United Kingdom** | **7** |
| - Cambridge | 4 |
| - Leeds | 3 |

## Table S3. Participant characteristics at randomization

|  | **Overall**  **(N=20)** | **Diluted first**  **(N=11)** | **Non-diluted first**  **(N=9)** |
| --- | --- | --- | --- |
| **Age (years)** |  |  |  |
| *median (IQR)* | 5 (3, 6) | 5 (3, 6) | 5 (4, 6) |
| *range* | 1 to 7 | 1 to 7 | 2 to 7 |
| **Gender – male** *n (%)* | 11 (55%) | 5 (45%) | 6 (67%) |
| **Race/ethnicity** |  |  |  |
| White | 17 (85%) | 10 (91%) | 7 (78%) |
| Asian | 1 (5%) | 1 (9%) | 0 (0%) |
| Mixed | 2 (10%) | 0 (0%) | 2 (22%) |
| **Diabetes duration (years)** |  |  |  |
| *mean ± SD* | 3.1 ± 1.5 | 2.9 ± 1.5 | 3.3 ± 1.6 |
| *range* | 0.5 to 5.8 | 0.5 to 5.5 | 1.3 to 5.8 |
| **Age-gender adjusted BMI percentile** *median (IQR)* ^a^ | 73% (43%, 87%) | 62% (55%, 75%) | 81% (34%, 87%) |
| **Glycated haemoglobin** |  |  |  |
| *mean ± SD (%)* | 7.4 ± 0.8 | 7.7 ± 0.8 | 7.0 ± 0.6 |
| *mean ± SD (mmol/mol)* | 57 ± 9 | 61 ± 9 | 53 ± 7 |
| *range (%)* | 5.9 to 8.7 | 6.3 to 8.7 | 5.9 to 7.6 |
| *range (mmol/mol)* | 41 to 72 | 45 to 72 | 41 to 60 |

^a^ Excludes one subject under two years of age who received diluted insulin first. For this subject, BMI percentile could not be calculated.
